# Predicting neuropsychological outcome following shunt operation in iNPH using reliable change indices (RCIs)

**DOI:** 10.1186/2045-8118-12-S1-O45

**Published:** 2015-09-18

**Authors:** Maria Wallin, Mats Tullberg, Carsten Wikkelsö, Per Hellström

**Affiliations:** 1 Hydrocephalus research unit at the institute of neuroscience and physiology, Sweden

## Introduction

While most iNPH patients experience a change in neuropsychological functioning after shunt operation there is a considerable variation in the extent, direction and nature of change. In this study we examined preoperative characteristics and their relationship to neuropsychological preoperative functioning and postoperative change. To determine neuropsychological change, reliable change indices (RCIs) were used. RCIs have been used to identify cognitive changes in other populations such as epilepsy, Parkinson's disease, dementia and sports medicine but have never previously been used in iNPH research. The aim of the study was to aid patients and healthcare professionals to make informed decisions regarding surgery and to introduce the use of RCIs to establish change after shunting.

## Methods

Data from 290 consecutive patients with iNPH that were referred to the Sahlgrenska university hospital iNPH unit between 1999 and 2013 were included in the analysis. These patients had undergone a brief neuropsychological assessment pre- and postoperatively. The test battery consisted of Rey Auditory Verbal Learning Test, Grooved Pegboard and Stroop. RCIs were used to quantify change. Multiple regression analysis was used to examine the effects of age, sex, education, amount of sleep, vascular risk factors, gait, balance, paratonia, and continence on preoperative neuropsychological function and RCI.

## Results

Improvement and deterioration in the different neuropsychological domains were determined using RCI with 80% confidence limit. The proportions of patients who improved were 26-47% and 6-18% deteriorated. The preliminary results show that patients with more pronounced preoperative iNPH symptomatology and vascular risk factors had a lower neuropsychological level of functioning. Female patients and younger patients with higher education tended to show slightly better results. The correlations between preoperative characteristics and RCIs were modest.

## Conclusions

RCI is a measurement that can detect and determine meaningful changes in iNPH patients after shunting. The measure has been successfully used in several other areas to identify cognitive changes after treatment. The introduction of RCI in iNPH research and clincal practice can improve the certainty of judgments pertaining to neuropsychological changes after shunting.

